# Systematic and evolutionary engineering of a xylose isomerase-based pathway in *Saccharomyces cerevisiae* for efficient conversion yields

**DOI:** 10.1186/s13068-014-0122-x

**Published:** 2014-08-20

**Authors:** Sun-Mi Lee, Taylor Jellison, Hal S Alper

**Affiliations:** McKetta Department of Chemical Engineering, The University of Texas at Austin, 200 E Dean Keeton Street, Stop C0400, Austin, TX 78712 USA; Clean Energy Research Center, Korea Institute of Science and Technology, 39-1 Hawolgok-dong, Seoul, 136-791 Seongbuk-gu Korea; Institute for Cellular and Molecular Biology, The University of Texas at Austin, 2500 Speedway Avenue, Austin, TX 78712 USA

**Keywords:** Xylose isomerase, Xylose fermentation, *Saccharomyces cerevisiae*, Adaptive evolution, Metabolic engineering

## Abstract

**Background:**

Efficient xylose fermentation by yeast would improve the economical and sustainable nature of biofuels production from lignocellulosic biomass. However, the efficiency of xylose fermentation by the yeast *Saccharomyces cerevisiae* is suboptimal, especially in conversion yield, despite decades of research. Here, we present an improved performance of *S. cerevisiae* in xylose fermentation through systematic and evolutionary engineering approaches.

**Results:**

The engineering of *S. cerevisiae* harboring xylose isomerase-based pathway significantly improved the xylose fermentation performance without the need for intensive downstream pathway engineering. This strain contained two integrated copies of a mutant xylose isomerase, *gre3* and *pho13* deletion and *XKS1* and *S. stipitis tal1* overexpression. This strain was subjected to rapid adaptive evolution to yield the final, evolved strain (SXA-R2P-E) which could efficiently convert xylose to ethanol with a yield of 0.45 g ethanol/g xylose, the highest yield reported to date. The xylose consumption and ethanol production rates, 0.98 g xylose g cell^−1^ h^−1^ and 0.44 g ethanol g cell^−1^ h^−1^, respectively, were also among the highest reported. During this process, the positive effect of a *pho13* deletion was identified for a xylose isomerase-containing strain and resulted in up to an 8.2-fold increase in aerobic growth rate on xylose. Moreover, these results demonstrated that low inoculum size and the cell transfer at exponential phase was found to be the most effective adaptation strategy during a batch culture adaptation process.

**Conclusions:**

These results suggest that the xylose isomerase pathway should be the pathway of choice for efficient xylose fermentation in *S. cerevisiae* as it can outperform strains with the oxidoreductase pathway in terms of yield and ethanol production and xylose consumption rates. Consequently, the strain developed in this study could significantly improve the prospect of biofuels production from lignocellulosic biomass.

**Electronic supplementary material:**

The online version of this article (doi:10.1186/s13068-014-0122-x) contains supplementary material, which is available to authorized users.

## Background

Lignocellulosic biomass can provide a sustainable feedstock for biofuels production. However, the inefficient fermentation of constituent pentose sugars such as xylose and arabinose limit the industrial-scale conversion of lignocellulose by the yeast *Saccharomyces cerevisiae* [1,2]. Several metabolic deficiencies in this yeast, including the lack of an endogenous pathway for xylose catabolism, require metabolic engineering. Many efforts have been reported that introduce heterologous xylose catabolism, such as the oxidoreductase pathway from *Scheffersomyces stipitis* (encoded by *xyl1*, *xyl2*, and *xyl3*) and isomerase pathway from *Piromyces sp.*, (encoded by *xylA*) [3,4]. While initial incorporation of these heterologous xylose catabolic pathways enable *S. cerevisiae* to convert xylose to ethanol, complementation is not enough and strains suffer from either low ethanol yield or productivity (or both) and thus require further improvement in xylose fermentation [2]. As a result, significant efforts have been made in modifying heterologous enzymes [5-7], optimizing metabolic flux through gene overexpression [8-11] or deletion [12-14], evolving xylose-utilizing strains by evolutionary engineering [15-18], and identifying improved xylose transporter proteins [19-21].

As examples of additional strain engineering, the overexpression of xylulokinase [9] and downstream genes involved in pentose phosphate pathway [11], and the deletion of *gre3* [14] or *pho13* [13,22] genes have been shown to significantly improve xylose utilization rates and efficiencies through reduced xylitol formation. Individual enzyme modifications to alter cofactor preference of xylose reductase and xylitol dehydrogenase [6,7], or improving enzyme activity of xylose isomerase by directed evolution [5], have improved heterologous xylose catabolism performance. Furthermore, whole-cell evolutionary engineering has been routinely applied to these rationally engineered strains to boost xylose fermentation efficiency [16-18]. Despite this array of attempts, the efficiency (especially yield) of xylose fermentation still remains suboptimal to achieve the goal of economical and sustainable biofuel production from lignocellulosic biomass, especially when compared with glucose conversion.

Until recently, the oxidoreductase pathway has been more intensely studied since consumption and growth rates with the xylose isomerase pathway have been extremely low [4]. However, the isomerase pathway does not require extensive cofactors as in the oxidoreductase pathway and thus has higher potential in terms of theoretical yield (0.51 g ethanol/g xylose) [5]. Toward this end, the experimental ethanol yields reported for the oxidoreductase pathway range from 0.09 to 0.39 for optimized strains [23,24], whereas reports as high as 0.43 g ethanol/g xylose can be found for the isomerase pathway [16,25]. Therefore, there is considerable interest in improving a xylose isomerase-based pathway in *S. cerevisiae* with a particular focus on improving both xylose consumption rates and yields [5,16,26].

Recently, our group subjected the xylose isomerase enzyme, *xylA* from *Piromyces sp.*, to directed evolution [5]. In doing so, a xylose isomerase mutant was identified that improved aerobic growth rate by 61-fold and both ethanol production and xylose consumption rates by 8-fold. These results were obtained with a minimally engineered strain of *S. cerevisiae* and achieved rates that were comparable with the oxidoreductase pathway. Here, we further improve xylose-isomerase based catabolism of xylose in *S. cerevisiae* by using a combination of rational and evolutionary engineering in a rapid fashion. As a rational engineering approach, we integrated two copies of the xylose isomerase mutant gene (xylA3*) into the genome of *S. cerevisiae* along with genomic overexpressions of native xylulokinase (*XKS1*) and heterologous transaldolase (*tal1*) from *S. stipitis*. We demonstrated that the deletion of *pho13* can significantly improve the cell growth on xylose using the isomerase pathway only in high-flux strains. Following this rational engineering, we subjected this strain to a rapid adaptation and obtained an evolved strain with the highest ethanol yield from xylose reported to date, coupled with the second highest ethanol production and xylose consumption rates reported.

## Results

### Rational construction of xylose isomerase-based strains

In this study, we sought to develop a *S. cerevisiae* strain with improved xylose catabolic rates and yields using the xylose isomerase pathway. To this end, we first established a genomic integration of the xylose isomerase pathway in *S. cerevisiae* by expressing a mutant xylose isomerase, xylA3*, developed by our group [5]. In our prior work, episomal expression of xylA3* combined with overexpression of the native *XKS1* and *tal1* from *S. stipitis* resulted in improved growth rates and xylose consumption rates [5]. To achieve efficient xylose utilization, we first integrated xylA3* into the genome of a *S. cerevisiae* BY4741 *gre3* knockout strain overexpressing *XKS1*, which was named as SXA-R1. In order to achieve catabolic levels commensurate with our previous plasmid-borne strain, an additional copy of xylA3* and *tal1* from *S. stipitis* were integrated into SXA-R1 generating SXA-R2 (Table 1).

**Table 1:** ***Saccharomyces cerevisiae*** **strains used in this study**

**Strains**	**Characteristics**
BY4741 Δ*gre3*	*Mat a, his3*Δ*1, leu2*Δ*0, met15*Δ*0, ura3*Δ*0, YHR104w::kanMX4*
BY4741 *Δpho13*	*Mat a, his3*Δ*1, leu2*Δ*0, met15*Δ*0, ura3*Δ*0, YDL236w::kanMX4,*
SXA-R1	BY4741 Δgre3, URA::GPDp-xylA*3-CYC1t-TEFp- XKS1-CYC1t
SXA-R2	BY4741 Δgre3, URA::GPDp-xylA*3-CYC1t-TEFp- XKS1-CYC1t, Leu:: GPDp-xylA*3-RPM1t-TEFp- tal1-CYC1t
SXA-R1P	SXA-R1, *YDL236w :: His*
SXA-R2P	SXA-R2, *YDL236w:: His*
SXA-R2P-E	Evolved strain of SXA-R2P

### The effect of *pho13* deletion with a xylose isomerase pathway

In an effort to further improve xylose consumption rates, we evaluated the impact of a *pho13* deletion on the xylose isomerase pathway. The *pho13* deletion has been reported to improve xylose fermentation and tolerance to toxic chemicals in lignocellulosic hydrolysate [12,13,27,28]. However, the improvement conferred by this deletion was mostly seen in strains harboring an oxidoreductase pathway with only marginally exhibited improvement with xylose isomerase-based strains [13]. We hypothesized that the improvement of a *pho13* deletion could have been muted in prior xylose isomerase strains due to low catabolic rates. Therefore, we investigated the effect of deleting *pho13* in strains harboring the mutant xylose isomerase pathways, which have higher xylose catabolic rates compared to wild-type xylose isomerase [5].

**Figure 1:**
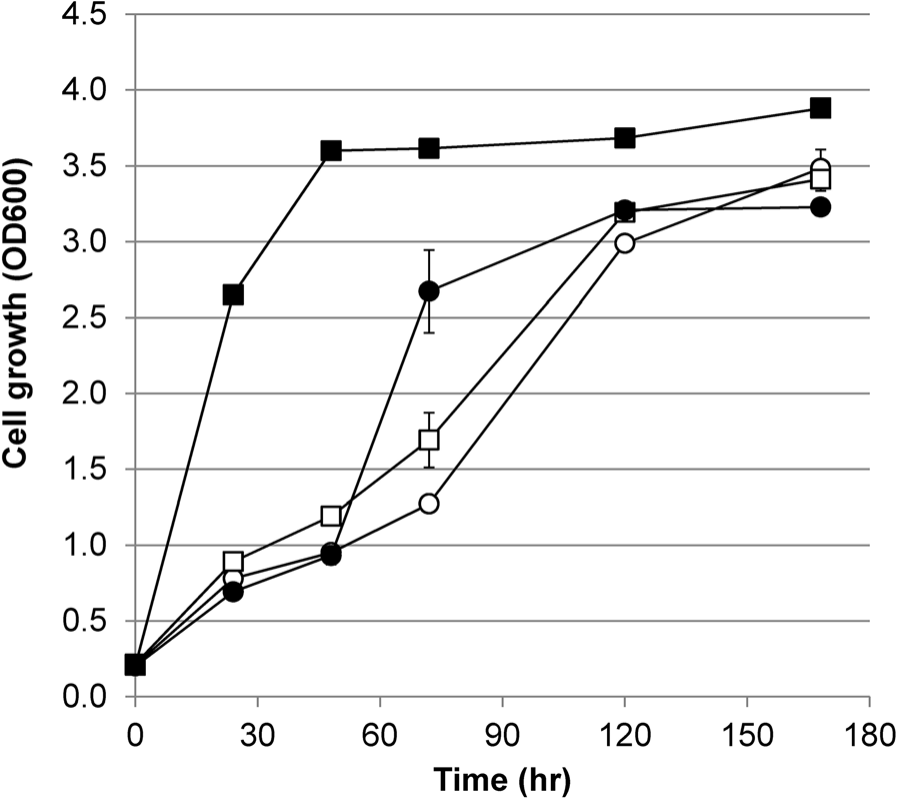
**Rational engineering of** ***S. cerevisiae*** **expressing a xylose isomerase pathway.** Rational strain engineering in a *gre3* background containing an integrated copy of *xylA*3* and *XKS1* overexpression (SXA-R1 strain) was evaluated. In addition, the expression of an additional copy of xylA3* and *tal1* overexpression (square), and *pho13* deletion (black) gradually improved the cell growth on xylose. Strain identifications (SXA-R1: white circle, SXA-R2: white square, SXA-R1P: black circle, and SXA-R2P: black square) are described in the text. The expression of an additional copy of xylA3* and *pho13* deletion showed synergistic effect on the cell growth on xylose. Error bars represent the standard deviation of biological triplicates.

Here, the effect of *pho13* deletion was investigated in strains expressing different levels of xylose isomerase (thus conferring different levels of cell growth). To this end, we deleted the *pho13* gene in the rationally engineered strains of SXA-R1 and SXA-R2 described above to make strains SXA-R1P and SXA-R2P, respectively. By deleting *pho13*, both rationally engineered strains showed significant increases in cell growth on xylose (Figure 1). As suspected, the impact of the *pho13* deletion was more pronounced in strains with a higher expression of xylose isomerase. Specifically, the *pho13* deletion increased aerobic growth rates 2.5-fold and 8.2-fold in SXA1-R1P and SXA-R2P respectively, compared to their respective controls. This result suggests that the beneficial effect of the *pho13* deletion is only clear when xylose isomerase expression is sufficiently high. This result could explain prior reports of *pho13* deletion being insignificant in xylose isomerase strains [29] supporting the importance of coupling high xylose isomerase pathway flux with a *pho13* deletion.

### Evolutionary engineering of the rationally engineered xylose-utilizing strain

Upon developing the rationally engineered strain SXA-R2P containing two copies of xylA3*, *gre3* and *pho13* deletions, and overexpression of XKS1 and *tal1*, we sought to further improve xylose catabolism in this strain by evolutionary engineering. Despite the effectiveness and simplicity of evolutionary engineering to improve desired phenotypes, there are no standardized methods for an effective adaptation process. To this end, we sought to evolve the rationally engineered strain SXA-R2P using serial subculturing in xylose medium and evaluate the success rate using different inoculum sizes and different cell growth phases. The inoculum sizes used in this study were 0.5, 1, and 5% and cell transfers into fresh medium were conducted when the cell growth was at exponential and stationary phases.

**Figure 2:**
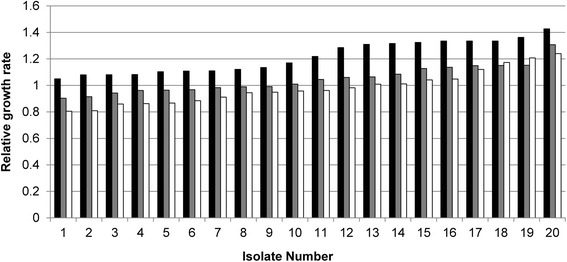
**Adaptive evolution of the rationally engineered strain expressing the xylose isomerase pathway.** The adaptive evolution of the rationally engineered strain (SXA-R2P) was performed in the exponential cell growth phase transfer set with different inoculum sizes of low (0.5%: black), medium (1%: grey), and high (5%: white). The graph shows representative strains with improved cell growth on xylose. The strains adapted in the culture with low inoculum size showed the highest improvement in the cell growth.

During the rapid 24-day adaptation process investigated here, cultures were transferred 12 times in the case of the exponential phase transfer set and five times in the case of the stationary phase transfer set. Overall adaptation rate was determined on the basis of growth rate advantage. As adaptation progressed, the cell growth rate on xylose increased progressively for the exponential phase transfer set. Most specifically, the most significant increase in the cell growth was observed from the set using low inoculum size. In contrast, the cultures from the stationary phase transfer set showed no significant growth adaptation, and the differences among various inoculum sizes were negligible. These data suggest that the choice of cell growth phase plays a significant role in obtaining growth advantage in batch culture adaptation as well as defining success for an evolutionary engineering experiment. Following this adaptation process, 130 evolved strains from the exponential phase transfer set were isolated and tested for growth on xylose by using Bioscreen C for high throughput analysis. The cell growth of 20 representative strains from each inoculum sizes are shown in Figure 2. The isolated strains from the pool with the low inoculum size (0.5%) showed highest improvement in the growth rates on xylose compared to those from the medium and high inoculum sizes (1% and 5%, respectively). As inoculum size increased, the efficiency in adaptation decreased, thus demonstrating the importance of this parameter in defining success for an evolutionary engineering experiment. Of these improved xylose-utilizing strains, the isolate showing the highest growth on xylose was selected as the evolved strain and designated as SXA-R2P-E. When evaluated in 14 ml culture tubes, SXA-R2P-E showed an aerobic growth rate of 0.128 h^−1^, which was 22% higher than that of the initial strain, SXA-R2P (0.105 h^−1^; Additional file 1: Figure S1). This growth rate is among the highest reported for a xylose isomerase-based strain.

### Improved xylose fermentation in the evolved strain SXA-R2P-E

**Figure 3:**
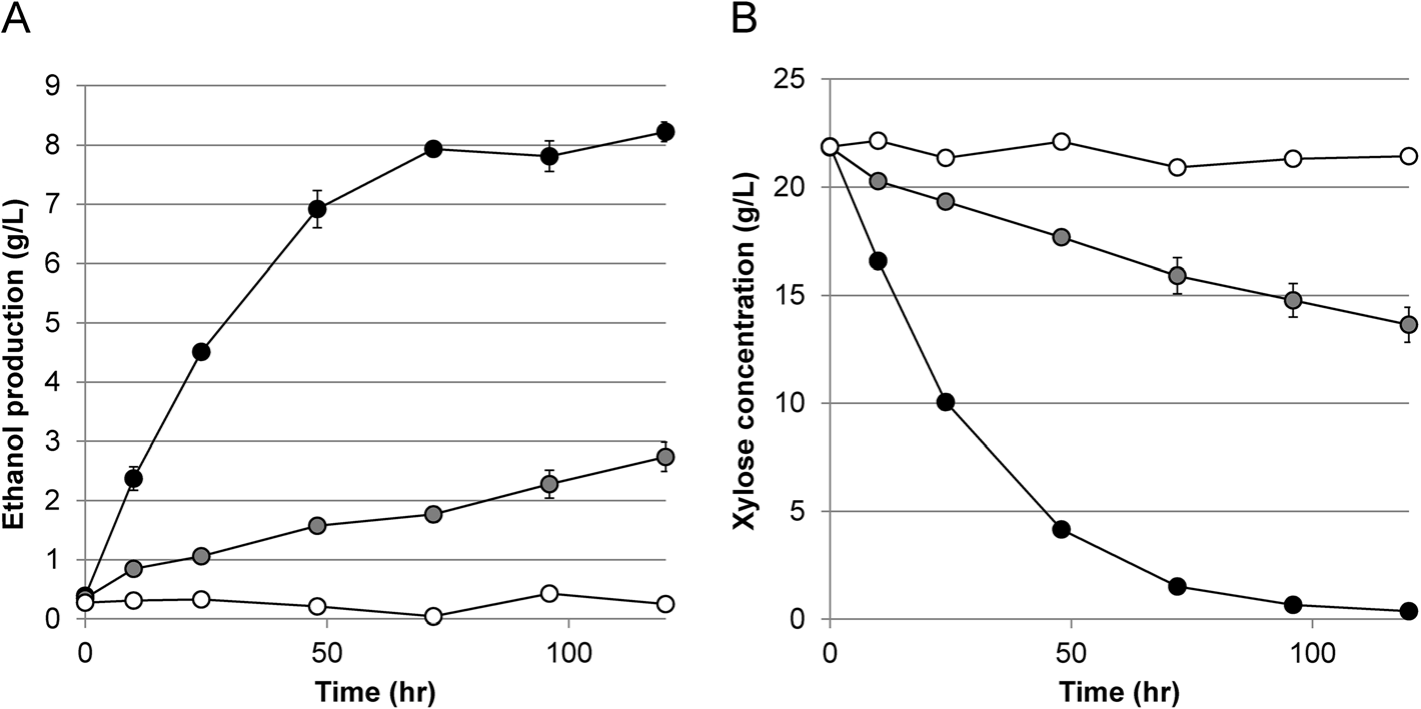
**Micro-aerobic fermentation tests with the evolved strain.** Ethanol production **(A)** and xylose consumption **(B)** profiles were measured in micro-aerobic conditions for wild-type (white), the rationally engineered strain (grey), and evolved strain (black) of *S. cerevisiae*. Ethanol production and xylose consumption were significantly increased in the evolved strain. Total improvement in both ethanol production and xylose consumption rates were about 4-fold. Error bars represent the standard deviation of biological triplicates.

To quantify the improvement of xylose fermentation performance of the evolved strain SXA-R2P-E, we performed high-cell density (OD = 20) batch fermentations in the micro-aerobic condition afforded by culturing 40 ml in a 50 ml sealed vial. Ethanol production capacity was greatly enhanced in SXA-R2P-E compared to the initial strain of SXA-R2P and the control strain, which does not have a xylose catabolic pathway (Figure 3A). After 72 hours of fermentation, SXA-R2P-E produced nearly 8 g/L of ethanol from about 20 g/L of xylose representing near complete consumption of xylose in this same timeframe (Figure 3B). The ethanol production and xylose consumption rates for this evolved strain (0.054 ± 0.002 g ethanol g cell^−1^ h^−1^ and 0.141 ± 0.003 g xylose g cell^−1^ h^−1^) were 3.9-fold and 4.3-fold higher than those of the starting strain SXA-R2P (0.014 ± 0.002 g ethanol g cell^−1^ h^−1^ and 0.033 ± 0.003 g xylose g cell^−1^ h^−1^). These increases were accompanied by an almost 20% increase in yield of ethanol approaching 0.39 g ethanol/g xylose for the evolved strain (Table 2). These results suggest that the adaptation process successfully improved the xylose catabolism in the rationally engineered strain and resulted in a strain with highly efficient xylose catabolism.

**Table 2:** **Comparison of representative previously reported xylose fermentation performances**

**Strains**	**Strain description**	**Conditions**	**Xylose consumption rate (g/g** ^**−1**^ **h** ^**−1**^ **)**	**Ethanol production rate (g/g** ^**−1**^ **h** ^**−1**^ **)**	**Ethanol yield (g/g** ^**−1**^ **)**	**Reference**
**SXA-R2P-E**	xylA*3, *tal1*, *XKS1*, *Δgre3, Δpho13* Evolved (24 days)	Anaerobic batch in bioreactor, synthetic medium (40 g/L xylose)	0.98	0.44	0.45	This study
**H131-A3-CS**	*xylA, xyl3, TAL1, TKL1, RPE1, RKI1,* evolved (160 days)	Anaerobic batch in bioreactor, syntheticmedium (40 g/L xylose)	0.94	0.40	0.43	[16]
**H131-A3-ALCS**	*xylA, xyl3, TAL1, TKL1, RPE1, RKI1,* evolved (160 days)	Anaerobic batch in bioreactor, synthetic medium (40 g/L xylose)	1.87	0. 77	0.41	[16]
**CEN.PK- RWB218**	*xylA, XKS1, TKL1, RPE1, RKI1, Δgre3,* evolved (70 days)	Anaerobic batch in bioreactor, synthetic medium (20 g/L xylose)	-	0.49 (projected)	0.41	[17]
**SR8**	Xyl1, xyl2, xyl3, *Δald6,* Evolved (no detailed information)	Anaerobic batch in flasks, synthetic medium (40 g/L xylose)	0.65	0.25	0.39	[24]
**CEN.PK2-1C -TMB 3424**	Xyl1, xyl2, *XKS1, GAL2, TKL1, RPE1, RKI1, Δgre3* Selected (70 days)	Anaerobic batch in bioreactor, synthetic medium (60 g/L xylose)	0.89	0.32	0.36	[6]
**SR8**	Xyl1, xyl2, xyl3, *Δald6,* Evolved (no detailed information)	Oxygen-limited batch in flasks, complete medium (40 g/L xylose)	0.87	0.28	0.31	[15]

Fermentation performance of *S. cerevisiae* SXA-R2P-E was compared with previously reported results for representative isomerase and oxidoreductase pathways.

**Figure 4:**
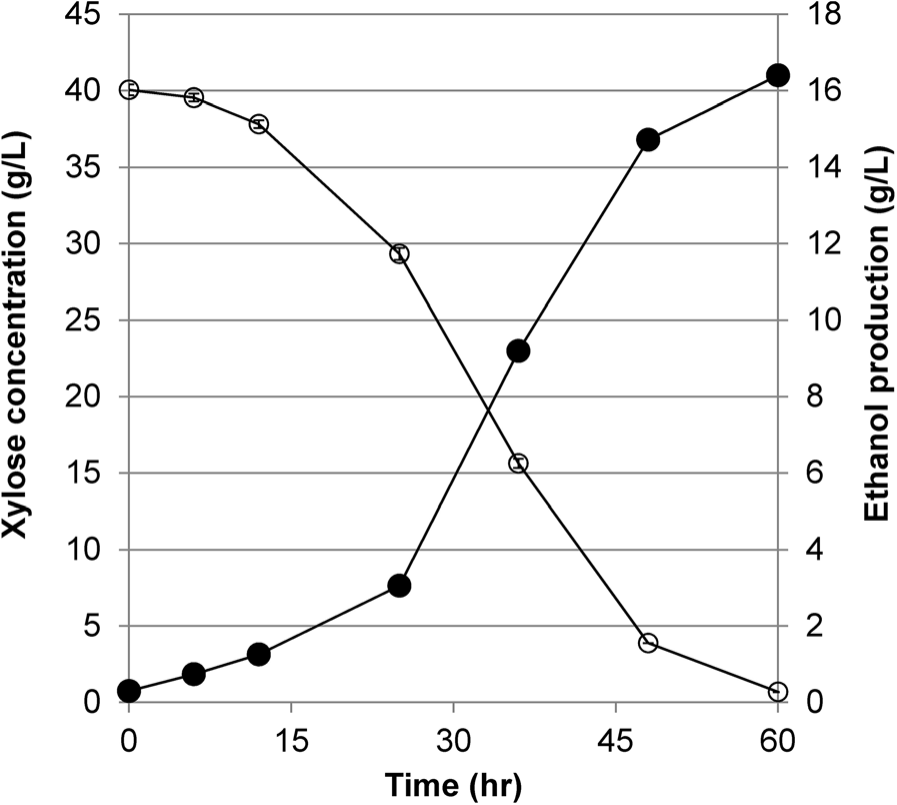
**Anaerobic fermentation of xylose with the evolved strain of SXA-R2P-E.** Ethanol production (black) and xylose concentration (white) profiles of the evolved strain were measured during anaerobic batch fermentation in a bioreactor. Error bars represent the standard deviation of technical duplicates.

To further improve the xylose fermentation performance and mimic more industrial-scale operation, we performed xylose fermentation in a bioreactor which offers more controlled fermentation conditions. The bioreactor was operated with no inlet airflow and minimal stirring to create an almost anaerobic condition after cells consumed all oxygen initially dissolved in the medium (typically around 12 hours as monitored by a dissolved oxygen sensor). Ethanol production from xylose was significantly improved under these conditions along with the xylose consumption rate (Figure 4). The ethanol production rate of SXA-R2P-E in the bioreactor reached 0.44 ± 0.01 g ethanol g cell^−1^ h^−1^, a value that is 8.1-fold higher than that obtained in the vial fermentation. The xylose consumption rate in the bioreactor also increased by 7.0-fold (0.98 g xylose g cell^−1^ h^−1^ versus 0.14 g xylose g cell^−1^ h^−1^ in the vial fermentation). Finally, these nearly anaerobic conditions and level of control resulted in a substantially increased ethanol yield of 0.45 g ethanol/g xylose, which is close to the theoretical yield value of 0.51 g/g [2]. Based on a survey of the literature, the xylose fermentation performance of SXA-R2P-E is the highest in terms of ethanol yield, and among the highest with respect to ethanol production and xylose consumption rates (Table 2).

## Discussion

This study reports on the development of a superior strain for xylose conversion into ethanol on the basis of yield, as well as xylose catabolic rate. To accomplish this feat, we combined a minimal set of rational engineering targets with short-term evolutionary engineering. The resulting ethanol yield (0.45 g ethanol/g xylose) was the highest reported to date, and ethanol production and xylose consumption rates were also among the highest reported.

Furthermore, we confirmed the beneficial effect of *pho13* deletion in a strain with a xylose isomerase pathway. In particular, we demonstrated that the *pho13* deletion was only beneficial when xylose isomerase was expressed at sufficient levels. These results are in contrast to a prior report that investigated the effect of *pho13* deletion in both a strain expressing an isomerase pathway (wild-type *xylA* gene), as well as a strain harboring the oxidoreductase pathway [29]. The beneficial effect of the *pho13* deletion was clear in the strain expressing an oxidoreductase pathway, but not for the isomerase pathway strain. Due to the inefficiency of a wild-type isomerase pathway [5], it might have been hard to confirm the effect of *pho13* deletion in this background. Here, we clearly demonstrate the beneficial effect of *pho13* deletion with an improved xylose isomerase pathway, especially when xylose isomerase is highly expressed (Figure 1). The aerobic growth rate of SXA-R2P was 8.2-fold higher than that of SXA-R2, whereas SXA-R1P showed only 2.5-fold higher aerobic growth rate compared to SXA-R1.

A secondary aspect of this work is the comparison of conditions for evolutionary engineering. In particular, we confirmed that inoculum sizes and cell growth phases were important parameters influencing the success of a rapid strain evolution project. Evolutionary engineering is a powerful approach to improve strains for a desired phenotype [30]. For the case of biofuel production, evolutionary engineering has led to strains with improved tolerances to products and the utilization of substrates [16,17,29,31]. However, the detailed information about effective adaptation processes is not available, nor are several conditions often compared. As a result, an adaptation process is usually conducted intuitively and takes a long period of time. In this study, we evaluated the effectiveness of rapid batch culture evolutionary engineering with respect to the conditions of inoculum sizes (low, medium, and high) and cell growth phases (exponential and stationary phases). Of the tested conditions, the most effective combination was the low inoculum size (0.5%) and exponential growth phase transfer - an easily understandable combination for rapid growth selection. In the end, the isolated strain showed an improved phenotype in a relatively short time frame of 24 days, and resulted in 4.3-fold increase in xylose consumption rate and 3.9-fold increase in ethanol production rate compared to the initial strain. It should be mentioned that we utilized a very rapid and effective method of evolutionary engineering. In contrast, for alternative work to improve *S. cerevisiae* expressing a xylose isomerase pathway, Kuyper *et al*. [17] and Zhou *et al*. [16] spent about 70 days and 160 days, respectively. Given the comparison between these strains (Table 2), it is evident that rapid evolutionary engineering combined with rational strain engineering can be quite effective for developing strains with improved xylose utilization.

This study presented a significantly improved xylose catabolism in *S. cerevisiae*. To our knowledge, the highest ethanol productivity and xylose consumption rates were reported by Zhou *et al*. [16] using a combinatorially engineered strain of wild-type xylose isomerase-expressing *S. cerevisiae* background with *xyl3, TAL1, TKL1, RPE1,* and *RKI1* overexpression (Table 2). Following the engineering of xylose catabolism, auxotrophic markers were restored and additional nutrients were supplemented, and this led significant improvement in the xylose fermentation performance, giving this strain the highest rates. Without the nutrient complementation, however, the performance of the combinatorially engineered strain itself (0.40 g ethanol g cell^−1^ h^−1^ and 0.94 g xylose g cell^−1^ h^−1^, and 0.43 g ethanol/g xylose) falls behind the values reported in this study (0.44 g ethanol g cell^−1^ h^−1^ and 0.98 g xylose g cell^−1^ h^−1^, and 0.45 g ethanol/g xylose). As a result, it can be presumed that optimizing culture condition or complementing nutrient requirement in SXA-R2P-E could enhance the xylose fermentation performance further. As an example, by changing the initial cell concentration and controlling to a different pH, SXA-R2P-E can achieve an even higher xylose consumption rate of 1.33 g xylose g cell^−1^ h^−1^ (see Additional file 2: Figure S2). In addition, it is possible that further rational genetic changes, including overexpression of downstream pentose phosphate pathway enzymes, could further improve the ethanol production and xylose consumption rates.

Additional changes notwithstanding, the results achieved in this study clearly show that the xylose isomerase pathway has distinct functional and theoretical advantage over an oxidoreductase pathway for xylose fermentation in *S. cerevisiae*. The low ethanol productivity and xylose consumption rates that have been major challenges in the field are alleviated. In particular, the strain developed here outperforms evolved strains expressing oxidoreductase pathways in terms of ethanol productivity, xylose consumption rates, and yields (last three rows of Table 2). The results obtained in this study and representative data from previous similar studies (summarized in Table 2) show that the ethanol yields obtained with an isomerase pathway are in the range of 0.41 to 0.45 g ethanol/g xylose, whereas those with an oxidoreductase pathway were in the range of 0.31 to 0.39 g ethanol/g xylose. The ethanol production and xylose consumption rates were also higher for an isomerase pathway which were in the range of 0.40 to 0.77 g ethanol g cell^−1^ h^−1^ and 0.98 to 1.87 g xylose g cell^−1^ h^−1^, respectively, compared to those for an oxidoreductase pathway (0.25 to 0.32 g ethanol g cell^−1^ h^−1^ and 0.65 to 0.89 g xylose g cell^−1^ h^−1^, respectively). Collectively, these results suggest that the xylose isomerase pathway should be the pathway of choice for efficient xylose fermentation in *S. cerevisiae*.

## Conclusions

This study presented a significantly improved xylose fermentation performance of *S. cerevisiae* expressing a xylose isomerase pathway along with minimal pathway engineering and evolutionary engineering. Without the need for extensive pathway engineering, the developed strain exhibits the highest ethanol yield (0.45 g ethanol g^-1^ xylose), and the second highest ethanol production and xylose consumption rates ever reported. During this process, the positive effect of *pho13* deletion in a strain with the xylose isomerase pathway was clearly demonstrated. This experiment shows that the xylose isomerase-based pathway as developed here should be the pathway of choice over the oxidoreductase pathway for xylose utilization in *S. cerevisiae*.

## Materials and methods

### Strains and culture conditions

*S. cerevisiae* strains used in this are summarized in Table 1. Yeast strains were routinely propagated at 30°C in yeast synthetic complete (YSC) medium composed of 6.7 g/L yeast nitrogen base, 20 g/L glucose, and CSM-Leu-Trp-Ura, CSM-Leu-His-Ura, or CSM-His-Leu-Trp-Ura (MP Biomedicals, Solon, Ohio, United States). Growth characterizations and ethanol fermentation were conducted in an identical medium except that 20 g/L or 40 g/L xylose was added as a carbon source. *Escherichia coli* strain *DH10β* was used for all cloning and plasmid propagation (New England Biolabs, Ipswich, MA, United States). *DH10β* was grown at 37°C in Luria-Bertani (LB) broth supplemented with 50 μg/mL of ampicillin (Sigma Aldrich, St. Louis, United States). All strains were cultivated with 225 rpm orbital shaking. Yeast and bacterial strains were stored at −80°C in 15% glycerol.

### Construction of xylose utilizing strain

To construct the xylose isomerase pathway, the xylose isomerase mutant (xylA3*) [5] under the control of the GPD promoter and an additional copy of *XKS1* under the control of the TEF promoter were integrated into the genome of *S. cerevisiae* BY4741 *gre3* knockout strain using integration vectors [19], resulting in SXA-R1. For the higher expression of xylose isomerase expression, an additional copy of xylA3* under the control of the GPD promoter, and *tal1* from *S. stipitis* under the control of the TEF promoter, were also integrated into genome of SXA-R1 strain, resulting in SXA-R2. The integration of the target genes was conducted by linearizing integration vectors by proper restriction enzymes and transforming into the target strains. Yeast transformation was conducted using Frozen EZ Yeast Transformation II Kit (Zymo Research, Irvine, California, United States) according to the manufacturer’s instructions. In each of SXA-R1 and SXA-R2 strains, the *pho13* gene was deleted to further improve the xylose utilization by homologous recombination. *Pho13* knockout strain of SXA-R1 and SXA-R2 were named as SXA-R1P and SXA-R2P, respectively.

### Evolutionary engineering of xylose utilizing strain

To improve xylose utilization of the rationally engineered strain, SXA-R2P was cultured and serial-transferred into 20 ml of fresh YSC medium with 20 g/L of xylose as a sole carbon source in closed 50 ml falcon tubes. The cells were transferred into fresh medium when they were at the exponential and stationary phase using 0.5, 1, and 5% inoculums in biological triplicates. The cells in the exponential phase transfer set were subcultured every 2 days with typical OD values in the range of 1.5 to 2.5. On the other hand, the stationary phase transfers were conducted every 5 days with typical OD values in the range of 2 to 3. After 12 and 5 rounds of exponential and stationary phase transfer, cells were plated onto YSC medium with 20 g/L xylose and the largest colonies were isolated. In total, 130 cells were selected from these plates for further characterization. Cell growth of isolated variants was first compared using Bioscreen C (GrowthCurvesUSA, Piscataway, NJ, USA) and then confirmed in 5 ml of YSC medium with 20 g/L of xylose in a 14 ml culture tube in biological triplicates. The fastest growing strain was selected and named as SXA-R2P-E.

### Growth analysis and ethanol fermentation

Aerobic growth rate analysis was performed at low yeast optical density while culturing cells at 30°C in 5 ml of YSC medium with 20 g/L of xylose in a 14 ml culture tubes. Cell density was measured spectrophotometrically at 600 nm absorbance, from which cell dry weight was calculated for the ethanol production and xylose consumption rates. Ethanol fermentations were performed in sealed 50 ml falcon tubes and a bioreactor (New Brunswick Scientific, Enfield, CT, United States). For small-scale fermentation, cells were grown at 30°C in 40 ml of YSC medium with 20 g/L of xylose in a sealed 50 ml falcon tube. For inoculum, one-day-old pre-culture (grown on glucose) was pelleted and re-suspended in xylose medium and inoculated at an initial OD of 20. For a large-scale experiment, ethanol fermentation was conducted in 3 L bioreactor (New Brunswick Scientific, Enfield, CT, United States) with 1.8 L of YSC medium with 40 g/L of xylose. The medium was supplemented with the anaerobic growth factors ergosterol (0.01 g/L) and Tween 80 (Sigma Aldrich, St. Louis, United States) (0.42 g/L) [16]. Medium pH was maintained at 5.0 with 2.5 N NaOH. Though the reactor was not purged with nitrogen gas, DO in the vessel was dropped after 12 hours of operation, and anaerobic condition was maintained throughout the fermentation. Xylose concentrations were measured using an YSI 7100 Multiparameter Bioanalytical System (YSI Life Sciences, Yellow Springs, Ohio, United States) and ethanol concentrations were measured using an Ethanol Assay, UV-method kit (R-Biopharm, Darmstadt, Germany). One OD_600_ unit was considered as 0.17 g cells/L [8]. Fermentation and growth assays were performed in biological triplicate except the fermentation performed in a bioreactor, which was assayed in technical duplicate.

## Additional files

Additional file 1: Figure S1. The aerobic cell growth of the evolutionary engineered strain of SXAR2P-E on xylose. The evolutionary engineering improved the aerobic cell growth of the strain harboring a xylose isomerase-based pathway on xylose. The evolved strain of SXA-R2P-E (black) reached stationary growth phase faster than the rationally engineered strain of SXA-R2P (grey). Error bars represent the standard deviation of biological triplicates. Additional file 2: Figure S2. Anaerobic fermentation of xylose with the evolved strain with low initial OD. Ethanol production (black) and xylose consumption (white) profiles of the evolved strain were measured during anaerobic batch fermentation in a bioreactor. The evolved strain was inoculated at an initial OD of 1. Medium pH was maintained at 6.0 with 2.5 N NaOH. Error bars represent the standard deviation of technical duplicates.

## Abbreviations

DO: Dissolved oxygen
g: Grams
h: Hours
L: Liter
OD or OD_600_: Optical density at 600 nm
