# Supplementary material for: Systematic and evolutionary engineering of a xylose isomerase-based pathway in Saccharomyces cerevisiae for efficient conversion yields
Source: Biotechnol Biofuels. 2014 Aug 20;7:122. doi: 10.1186/s13068-014-0122-x (PMC4147937; doi:10.1186/s13068-014-0122-x)

Supplementary Figure 1. *The aerobic cell growth of the evolutionary engineered strain of SXA-R2P-E on xylose.* The evolutionary engineering improved the aerobic cell growth of the strain harboring a xylose isomerase-based pathway on xylose. The evolved strain of *SXA-R2P-E* (black) reached stationary growth phase faster than the rationally engineered strain of SXA-R2P (grey). Error bars represent the standard deviation of biological triplicates.

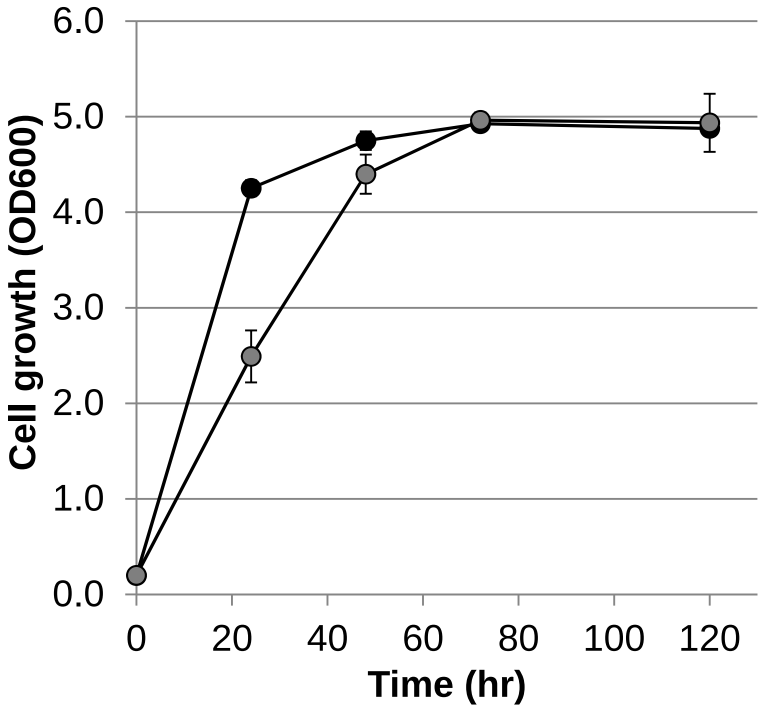

Supplement: Additional file 1: Figure S1. — The aerobic cell growth of the evolutionary engineered strain of SXAR2P-E on xylose. The evolutionary engineering improved the aerobic cell growth of the strain harboring a xylose isomerase-based pathway on xylose. The evolved strain of SXA-R2P-E (black) reached stationary growth phase faster than the rationally engineered strain of SXA-R2P (grey). Error bars represent the standard deviation of biological triplicates. [file 13068_2014_122_MOESM1_ESM.pdf]
