# Supplementary material for: Systematic and evolutionary engineering of a xylose isomerase-based pathway in Saccharomyces cerevisiae for efficient conversion yields
Source: Biotechnol Biofuels. 2014 Aug 20;7:122. doi: 10.1186/s13068-014-0122-x (PMC4147937; doi:10.1186/s13068-014-0122-x)

Supplementary Figure 2. *Anaerobic fermentation of xylose with the evolved strain with low initial OD.* Ethanol production (black) and xylose consumption (white) profiles of the evolved strain were measured during anaerobic batch fermentation in a bioreactor. The evolved strain was inoculated at an initial OD of 1. Medium pH was maintained at 6.0 with 2.5 N NaOH. Error bars represent the standard deviation of technical duplicates.

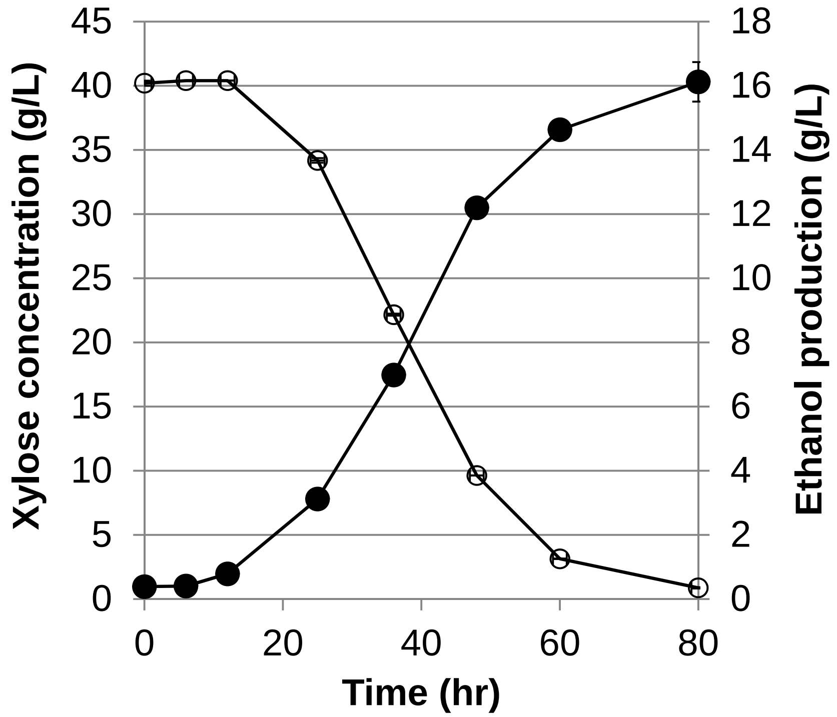

Supplement: Additional file 2: Figure S2. — Anaerobic fermentation of xylose with the evolved strain with low initial OD. Ethanol production (black) and xylose consumption (white) profiles of the evolved strain were measured during anaerobic batch fermentation in a bioreactor. The evolved strain was inoculated at an initial OD of 1. Medium pH was maintained at 6.0 with 2.5 N NaOH. Error bars represent the standard deviation of technical duplicates. [file 13068_2014_122_MOESM2_ESM.pdf]
